# GRIM-19 in asthenozoospermia regulates GC-2 spd cell proliferation, apoptosis and migration

**DOI:** 10.1038/s41598-023-29775-7

**Published:** 2023-02-22

**Authors:** Fei Li, Aiqin Niu, Kangjun Zhao, Jianbing Feng, Ying Chen

**Affiliations:** 1 Center for Reproductive Medicine, The First People’s Hospital of Shangqiu, 292 Kaixuan South Road, Shangqiu, Henan China; 2 The First Clinical College of Hubei University of Medicine, Hubei, China; 3 The First Affiliated Hospital of Zhengzhou University, Henan, China

**Keywords:** Diseases, Medical research, Urology

## Abstract

Asthenozoospermia (AZS) is a severe form of male infertility with no clear pathogenesis, despite numerous research efforts, there is no consensus on this. This study was to investigate the expression of gene-associated with retinoid-interferon-induced mortality 19 (GRIM-19) in the sperm of patients with asthenozoospermia and the regulation of GC-2 spd cell proliferation, apoptosis and migration. We analyzed the sperm samples from 82 asthenozoospermia and normal patients were collected in the First People's Hospital of Shangqiu and the First Affiliated Hospital of Zhengzhou University. Immunofluorescence, western blots and RT-qPCR analyses were used to verify the expressions of GRIM-19. MTT assays were used to assess cell proliferations, flow cytometry was performed to assess cell apoptosis, wound‑healing was performed to measure cell migration. Immunofluorescence showed that GRIM-19 is predominantly expressed in the sperm mid-piece, the mRNA expressions of GRIM-19 in sperms of the asthenozoospermia group were significantly low, relative to the normal group (OR 0.266; 95% CI = 0.081–0.868; *P* = 0.028). The protein expressions of GRIM-19 in sperms of the asthenozoospermia group were significantly lower than that of the normal group as well (GRIM-19/GAPDH: 0.827 ± 0.063 vs 0.458 ± 0.033; *P* < 0.001). GRIM-19 overexpression promotes GC-2 spd cell proliferation and migration and reduces apoptosis, while GRIM-19-silenced reduces GC-2 spd cell proliferation and migration and increased apoptosis. GRIM-19 is closely related to the occurrence of asthenozoospermia and promotes GC-2 spd cell proliferation and migration and reduces apoptosis.

## Introduction

Infertility is a global social concern since approximately 40–50% of infertility in couples is caused by male factors^1^. Asthenozoospermia is a common cause of male infertility characterized by reduced forward motility, the etiology and regulation mechanism of asthenozoospermia are still not fully understood^2,3^. In the past decades, some studies have been focused on studying the regulation mechanisms of asthenozoospermia, although several genes, signaling pathways has been found to be vital for asthenozoospermia, but there are contentions^4,5^.

Compared to normal individuals, asthenozoospermia patients exhibit significantly higher rates of sperm apoptosis and significantly lower proliferation as well as migration abilities. Sperm motility is dependent on energy availability, fully functioning flagellum, and interactions of several signaling pathways^6–8^. Previous studies have revealed that oxidative stress and the occurrence of asthenozoospermia are closely linked, elevated oxidative stress levels due to reactive oxygen species and related pathways results in abnormal sperm tail fiber sheath and mitochondrial structures, leading to decreased adenosine triphosphate levels and suppressed sperm flagellar movement abilities^9,10^. In the past few decades, human sperm quality and quantity have declined, therefore, elucidation of gene expression differences and regulation mechanisms of asthenozoospermia is of significance for comprehensive understanding and clinical treatment.

The gene-associated with retinoid-interferon-induced mortality 19 (GRIM-19) was first reported on chromosome 19p13.1 by Angell et al.^11^. It is widely expressed in the nucleus and cytoplasm of cells, and it is the basic functional subunit of mitochondrial nicotinamide adenine dinucleotide dehydrogenase complex (NADH), maintains normal functions of oxidative phosphorylation of the respiratory chain in the mitochondrial intima, and plays various roles in cell proliferation, apoptosis as well as regulation of different signaling pathways^12,13^. The mitochondria provide energy to the sperm via glycolysis and oxidative phosphorylation, therefore, once mitochondrial structure or function is impaired, the sperm loses its vitality^6,14^. GRIM-19 has an important role in cell electron transfer activities, studies have demonstrated down-regulation of GRIM-19 leads to the breakdown of mitochondrial membrane potential and increased reactive oxygen species (ROS) levels^15,16^, changes in ROS concentrations are closely associated with sperm motility, energy acquisition and acrosome reactions, which may suggest that GRIM-19 is closely related to the occurrence of asthenozoospermia.

In view of this, we hypothesized that GRIM-19 plays an important role in the occurrence and development of asthenozoospermia. In this study, we evaluated GRIM-19 levels in asthenozoospermia and investigated the roles of GRIM-19 in cell proliferation, migration as well as apoptosis. We expected that this study can reveal the role of GRIM-19 in asthenozoospermia.

## Materials and methods

### Ethical approval and informed consent

This study strictly followed the relevant requirements of the Declaration of Helsinki of the World Medical Association. Ethical approval was granted by the Medical Ethics Committee of Zhengzhou University (202036000Z120277). Informed consent was waived due to retrospective design of the study.

### Participants

Patients treated at the First People's Hospital of Shangqiu and the First Affiliated Hospital of Zhengzhou University between October 2020 and June 2021 served as subjects of the study. 41 asthenozoospermia patients (grade A + B sperm < 32%) were included in the study, along with 41 participants with normal semen parameters. The males were masturbated after 3–7 days of sexual abstinence. Semen samples were analysed immediately on delivery to the laboratory, using the computer-aided semen analysis (CASA) system (WLJY-9000; Beijing Weili New Century Science & Tech Dev Co, Ltd., China). Inclusion criteria: (1) patients aged between 20 and 45 years old with complete clinical data; (2) the chromosome karyotype is completely normal; (3) AZF gene test did not contain any deletion; (4) no syphilis, hepatitis, AIDS and other infectious diseases. Exclusion criteria: (1) patients with acute infection of the genitourinary system; (2) recently taking drugs that interfere with the quality of sperm or semen; (3) malignant tumor or abnormal liver and kidney function; (4) missing clinical data or medical records.

### Immunofluorescence

Cells localization of GRIM-19 in sperm was observed by immunofluorescence analysis. The prepared spermatozoa samples were smeared onto microscope slides, and Place at 37 °C for 15 min to dry. After that, the samples were fixed in phosphate-buffered saline (PAN-Biotech GmbH, Aidenbach, Germany) for 30 min with 4% paraformaldehyde (Yanmu Industrial Co. China), 0.2% Triton X-100 (Sigma–Aldrich, St Louis, MO, USA) was added and incubated for 15 min, then antibody dilution of GRIM-19 was added and sealed under a shaker at 4 °C. After blocking with goat serum diluted in PBS (Beyotime, Shanghai, China) for 1 h, slides were incubated overnight with ezrin mAb (1:250) and rabbit cd44 antibody (1:100) (ZhongShan Biotechnology Co. China). Slides were washed with PBS after TRITC (Tetramethylrhodamine-5-[and 6]-isothiocyanate)-conjugated goat anti-mouse for 1 h. After washing the slides in PBS and covering them with coverslips, the slides were examined using a LEICA TCS SPE confocal microscope (LEICA, Germany). Pictures were processed and analyzed using specialized software (Discus software, Germany).

### Cell lines and culture

Cells used in this study were GC-2 spd mouse spermatocytes (ATCC, Manassas, Virginia, USA). Culture conditions were: low-sugar DMEM (Manassas, VA, USA) medium + 1% double antibiotics (100 ug/ml penicillin + 100 ug/ml streptomycin) (North China Pharmaceutical Group Corp.China) + 10% fetal bovine serum medium at 37 °C in a sterile 5% CO_2_ environment. We purchased siRNA-GRIM-19 from Abcam Inc (UK), and pEGFP-GRIM-19 was purchased from Sangon Biotech (Shanghai, China).

### Sperm RNA extraction

Spermatozoa were purified by percoll discontinuous density gradient centrifugation, and the concentration of percoll separation solution is 25% and 50%, respectively. RNA extraction performed using the RNeasy Mini Kit (Qiagen Sciences, USA), as instructed by the manufacturer. Samples were placed in an enzyme-free Eppendorf (EP) tube and 300 μl Buffer RLT added to fully lyse the sperms. Then, 300 μl of 70% ethanol (Chemical Reagent, Guangzhou, China) were added into an EP tube without an enzyme. The 600 μl suspension was transferred to the RNeasy MinElute spin column, which was placed in a 2 ml enzyme-free collection tube. The tube was closed and centrifuged at 4 °C and 12,000 g for 15 s. The adsorption column was transferred to a new 2 ml collection tube, and 20 μl of RNA-free water (Thermo Fisher Scientific, USA) added to the filter membrane of the adsorption column. Then, centrifugation was done at 12,000 g for 2 min. The bottom liquid in the tube was the total RNA extract. Total RNA was extracted from GC-2 spd cells after which RT-qPCR was performed using the QuantiNova SYBR Green PCR Kit (Qiagen Sciences, USA). GRIM-19 primers were as follows: forward primer: 5′-TCGGGGACTGTCGGGGTAC-3′, Reverse-5′- AGGGTCCTCCGGTCCTTCT-3′.

### Western blot analysis

Sperm cells were lysed and centrifuged at 12,000 RPM at 4 °C for 10 min. SDS-PAGE protein loading buffer (FcMACS, NanJing, China) was added, mixed and centrifuged, followed by a metal bath at 100 °C for 5–10 min. Then, 15% PAGE separation glue (Aspen, Guangzhou, China) was prepared, thoroughly mixed and added to the glue-making glass plate. The upper glue was prepared after which the separation glue was carefully added to the upper layer along the edge of the glass plate, which was inserted into the sample comb and left to stand at room temperature until solidification of the glue. The prepared glass plate was fixed in the electrophoresis tank and electrophoresed for 25 min at a constant pressure of 70 V. After electrophoresis, the glass plate was opened, the gel was taken out, a PVDF membrane (Pall Life Sciences, US) of an appropriate size cut according to the gel for electrical transfer, and closed. After sealing, the blots were washed 3–5 times using TBST (ZSGB-BIO, Beijing, China) and were treated with specific primary antibodies overnight. The blots were cut according to the size of target proteins prior to hybridization with primary antibodies, the secondary antibodies were then used to incubate the blots (ZSGB-BIO, Beijing, China) and an ECL (Share Bio, Shanghai, China) was applied to visualize the blots.

### Transfection of GC-2 spd cell with siRNA-GRIM-19

GC-2 spd cell were inoculated on 6-well plates at a density of 2–5 × 10^5^/ml. siRNA transfected GC-2 spd cell were divided into 2 groups, for the siRNA-GRIM-19 (Santa Cruz Biotechnology, CA, USA) and control groups, 3 multiple holes were set for both groups. siRNA 5 nmol and RNA enzyme-free (Fermentas Life Sciences, USA) water 250 μl were mixed to prepare an siRNA stock solution at a concentration of 20 μmol/l. The stock solution was separately stored in an ultra-low temperature refrigerator. Then, 7.5 μl Lipofectamine 2000 (Thermo Fischer, USA) was added to a 1.5 ml centrifugation tube and supplemented with serum-free medium to 125 μl, as instructed by the manufacturer. An siRNA liposome complex (250 μl) was inoculated into 6-well plates after which 2 ml of culture medium containing FBS (Manassas, VA, USA) and double antibody (North China Pharmaceutical Group Corp. China) was added to each well. Incubation was done in a 5% CO_2_ environment at 37 °C for 24 h.

### Transfection of GC-2 spd cell with pEGFP-GRIM-19

GC-2 spd cell were inoculated on 6-well plates at a density of 2–5 × 10^5^/ml. Transfection was performed at 70–80% confluence, then, cells were divided into PEGFP-GRIM-19 (Santa Cruz Biotechnology, CA, USA) and control groups, and 3 multiple holes were set for both groups. About 7.5 μl of Lipofectamine 2000 (Thermo Fischer, USA) was transferred into a 1.5 ml centrifuge tube and serum-free medium added to 125 μl. Plasmid DNA (Omega, USA) 10 μl was transferred to a 1.5 ml centrifuge tube and supplemented with 10 µl Lipofectamine 2000 and serum-free medium to prepare a plasmid DNA mixture. The volume was fixed to 250 μl. The plasmid DNA mixture was transferred to 6-well plates and supplemented with 2 ml of a medium containing FBS (Manassas, VA, USA) and double antibody (North China Pharmaceutical Group Corp, China). Incubation was done in a 5% CO_2_ incubator at 37 °C for 6 h.

### Evaluation of the proliferation levels of GC-2 spd cell

After counting the GC-2 spd cell, their concentration was adjusted to 1–5 × 10^5^/ml. Cells at 1–5 × 10^5^/ml were inoculated in 96-well plates at 100 μl/well after which they were incubated at 37 °C in a 5% CO_2_ atmosphere for 24 h. Then, 10 μl of 5 mg/ml MTT solution (Sigma, California, USA) was supplemented to each well and incubated for 4–6 h. The culture solution was discarded and 150 ul of dimethyl sulfoxide (Amresco, USA) added to each well and shaken for 10–15 min on a low-speed shaker to fully dissolve it. Absorbance of each well at 490 nm was read using a multifunctional enzyme-linked immunoassay. The proliferations of GC-2 spd cell in each group were calculated according to the OD value.

### Determination of apoptosis levels of GC-2 spd cell

GC-2 spd cell were inoculated in 6-well plates and incubated. Cells were washed 2–3 times using PBS (Beyotime, Shanghai, China), treated with an appropriate amount of trypsin, transferred to centrifuge tubes, centrifuged at 1000 g for 5 min and the supernatant discarded. The Annexin V-FITC conjugate (Beyotime Biotechnology, Shanghai, China) (200 μl) was added to resuspend the cells, incubated for 10 min at room temperature away from light, centrifuged at 200 g for 5 min, and the supernatant discarded. Subsequently, 190 μl of Annexin V-FITC conjugate and 10 μl of propidium iodide ((Beyotime Biotechnology, Shanghai, China)) staining solution were added, gently mixed, and placed in an ice bath away from light. Apoptosis levels were finally determined by flow cytometry.

### Analysis of the migration ability of GC-2 spd cell

The GC-2 spd cell were digested with trypsin (Sangon Biotech, Shanghai, China), inoculated into 6-well plates (5 × 10^5^ cells/well) and incubated at 37 ℃ in a 5% CO_2_ incubator for 24 h. When cells were spread all over the bottom of the culture dish, a straight line was drawn in the middle of the culture dish using a sterile 20 μl gun tip, perpendicular to the cells plane. After scratching was complete, cells were washed thrice using PBS (Beyotime, Shanghai, China), to remove non-adherent cells and exfoliated cells. The serum-free medium (Invitrogen, Carlsbad, CA, USA) was replaced and further incubation done at 37 °C in a 5% CO_2_ incubator. Healing of the scratch was observed and imaged by microscopy. Image J software was used to determine cell migration from the wound closure area, firstly, the scale of the cell scratch pictures was calibrated, and then the wound closure area was analyzed with the cell scratch pictures (initial scratch width- final scratch width) /initial scratch width × 100%. After the experimental data are obtained, R language analysis software is used for analysis and comparison.

### Statistical analysis

All analyses were performed using the Statistical Package for Social Sciences (Version 22.0; SPSS, Chicago, IL).and the statistical packages R (The R Foundation; http://www.r-project.org; version 3.6.1). Continuous variables were expressed as means ± standard deviation. Student’s t test or chi-square test were used for comparisons of means between groups. The Wilcoxon ran-sum test were used to test the nonparametric values. Differences were significant at p < 0.05.

## Results

### Baseline participant data

A total of 82 participants were included in this study; 41 cases in the asthenozoospermia group and 41 cases in the normal control group. There were no significant differences between the two groups in terms of age, years of infertility, body mass index, testicular size and semen volume, however, there were significant differences in sperm density, total sperm motility, percentage of immobile sperm and percentage of normal sperm (Table 1).

**Table 1:** Clinical characteristics of study participants.

Projects	Asthenozoospermia group (n = 41)	Normal control group (n = 41)	t/χ^2^ value	*p* value
Age (years)	30.6 ± 3.4	29.9 ± 3.3	0.946	0.349
Infertility years	4.5 ± 3.4	4.4 ± 3.7	0.213	0.834
Body Mass Index (kg/m^2^)	23.2 ± 4.3	23.2 ± 3.1	0.623	0.514
Testicular size (ml)	14.8 ± 5.2	14.1 ± 6.1	0.559	0.581
Semen volume (ml)	2.5 ± 1.8	3.0 ± 2.1	− 1.829	0.071
Semen PH value	7.3 ± 0.3	7.4 ± 0.2	− 1.754	0.083
sperm density (10^6^/ml)	40.5 ± 22.1	72.2 ± 41.7	− 4.248	< 0.001
forward progression of sperm (PR, %)	10.8 ± 5.3	39.2 ± 20.2	− 8.086	< 0.001
immobile sperm (%)	72.8 ± 32.9	35.0 ± 14.1	6.679	< 0.001
percentage of normal sperm (%)	2.5 ± 1.4	9.5 ± 3.3	− 12.350	< 0.001

The data were expressed as means ± standard deviations (SD), *p* < 0.05, statistically significant difference.

### Distribution of GRIM-19 in human sperm

**Figure 1:**
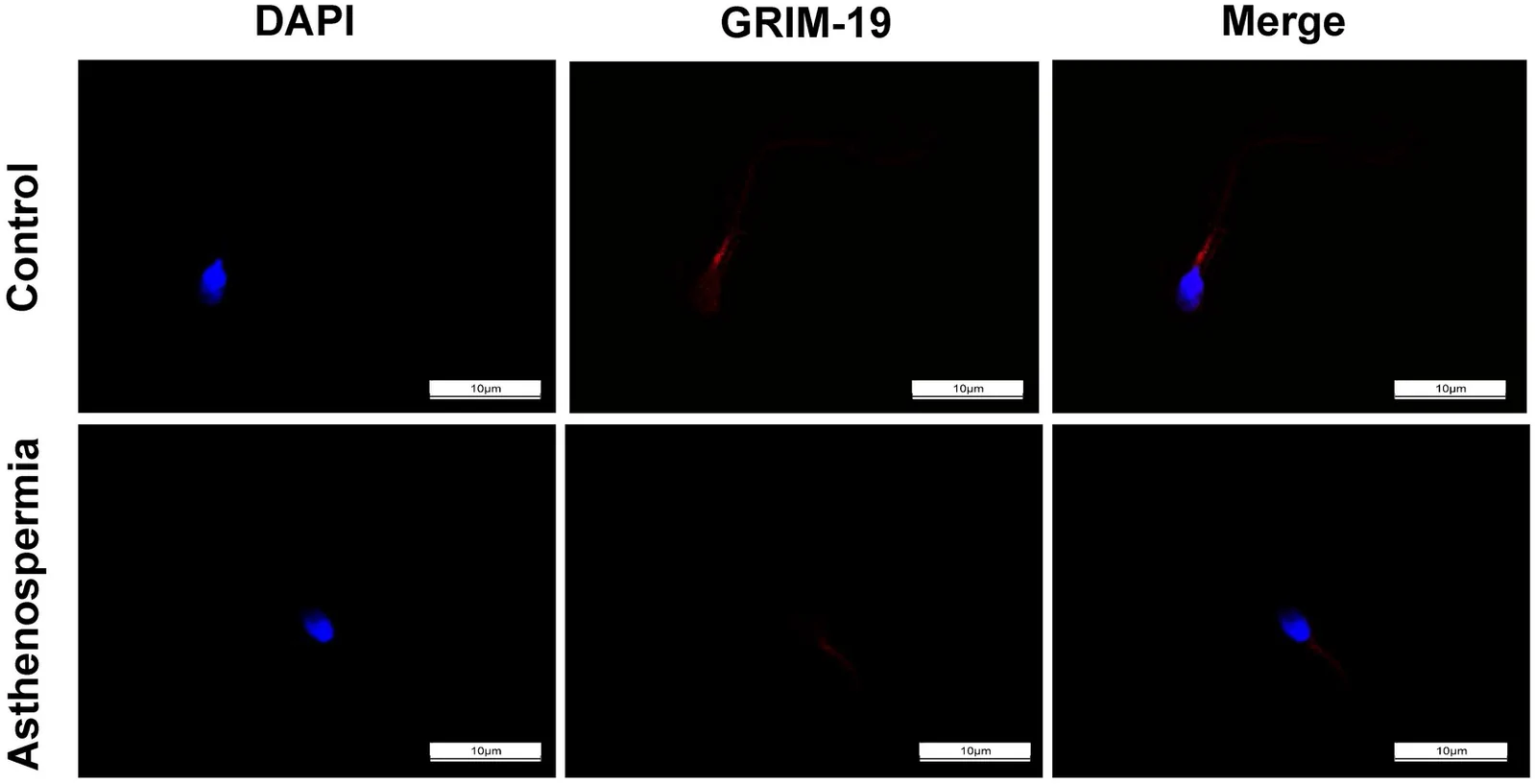
Immunofluorescent staining was carried out to investigate the subcellular localization of GRIM-19 in the sperm. DAPI (4′,6-diamidine-2-phenylindole) staining is shown on the left, middle column is GRIM-19 staining, the merge image is shown on the right.

Immunofluorescence was used to detect the distribution and expression position of GRIM-19 in sperm. The results showed that GRIM-19 was mainly distributed in the head and middle of sperm. In addition, by comparing the immunofluorescence intensity, we found that the expression of GRIM-19 in patients with asthenozoospermia was lower than that in patients with normal sperm (Fig. 1).

### Expressions of GRIM-19 in human sperm

**Figure 2:**
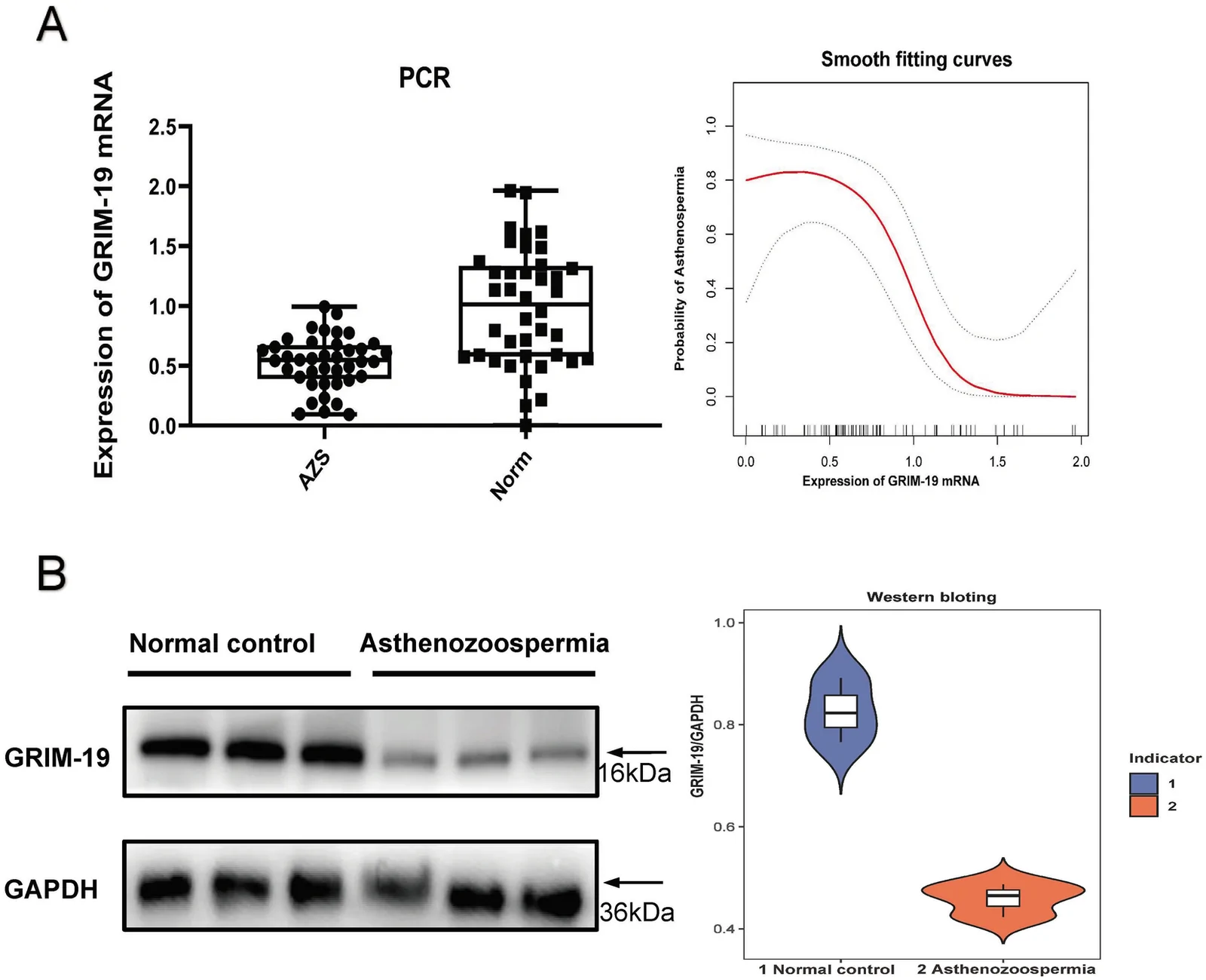
(**A**) shows the expressions of GRIM-19 protein in the normal control group and the asospermia group (p < 0.001). (**B**) shows the mRNA expressions of GRIM-19 as detected by RT-QPCR in the normal control and asthenozoospermia groups (p < 0.001).

The expression of mRNA of GRIM-19 in the spermatozoa of patients with asthenozoospermia was significantly lower than in the normal control group, the smooth-fitting curve further suggested that there was a negative correlation between GRIM-19 and asthenozoospermia (OR 0.266; 95% CI = 0.081–0.868; *P* = 0.028) (Fig. 2A). Western blotting analysis showed that the expression of protein of GRIM-19 in the spermatozoa of patients with asthenozoospermia was also significantly lower than in the normal control group (GRIM-19/GAPDH: 0.827 ± 0.063 vs 0.458 ± 0.033; *P* < 0.001) (Fig. 2B).

### GRIM-19 overexpression and silencing in GC-2 spd cell

**Figure 3:**
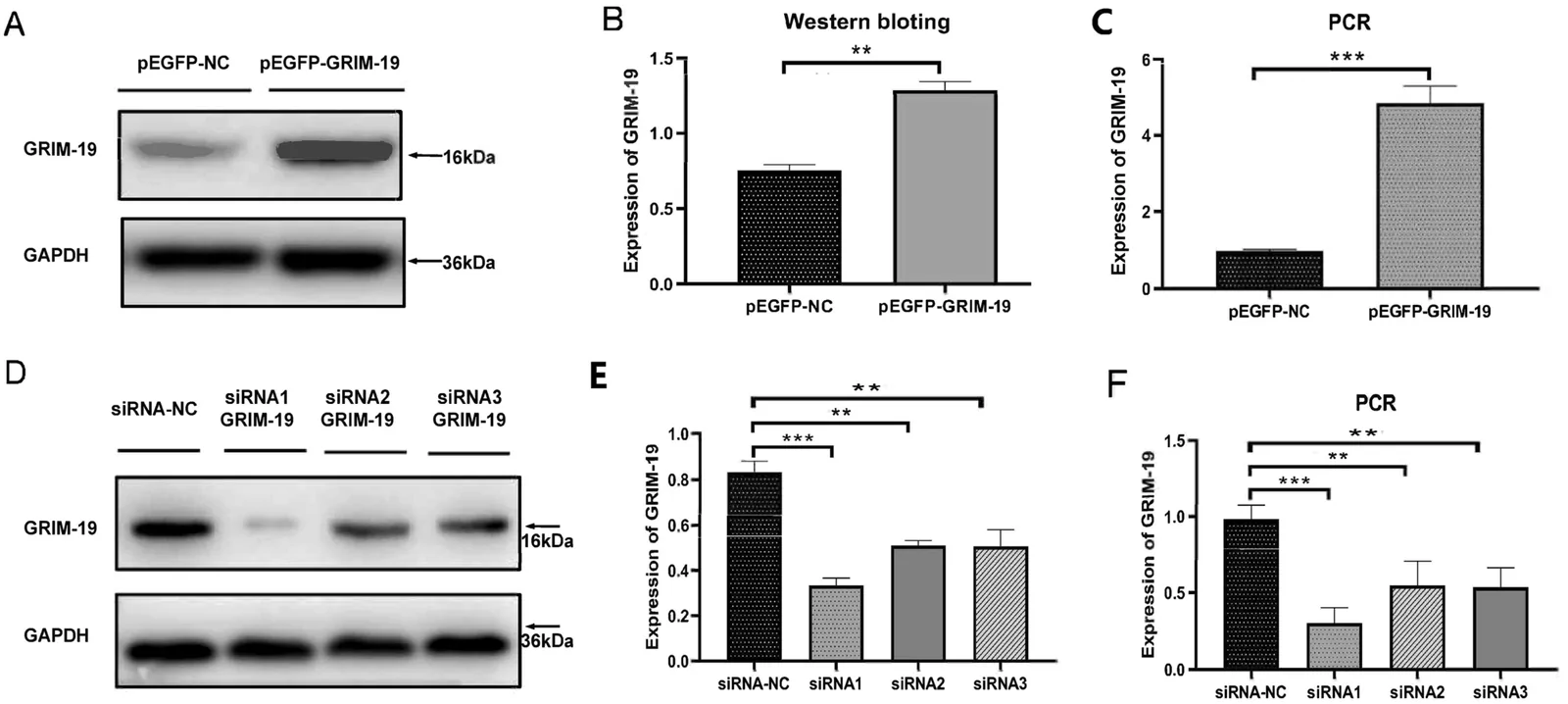
Expressions of GRIM-19 in GC-2 spd cell with overexpressed and silenced GRIM-19. (**A**) Western blotting analysis of protein expressions of GRIM-19 in pEGFP-NC and pEGFP-GRIM-19 cells; (**B**) Gray value analysis of western blotting results (pEGFP-NC vs pEGFP-GRIM-19); (**C**) RT-QPCR analysis of mRNA expression of GRIM-19 in pEGFP-NC and pEGFP-GRIM-19 cells; (**D**) Western blotting quantitative analysis of total protein expressions of GRIM-19 in siRNA-NC, siRNA1-GRIM-19, siRNA2-GRIM-19 and siRNA3-GRIM-19 cells; (**E**) Gray value analysis of western blotting results (siRNA-NC vs siRNA1-GRIM-19, siRNA2-GRIM-19, siRNA3-GRIM-19). (**F**) RT-QPCR analysis of mRNA expression of GRIM-19 in siRNA-NC, siRNA1-GRIM-19, siRNA2-GRIM-19 and siRNA3-GRIM-19 cells. ** represents p < 0.01, *** represents p < 0.001, NC represents Natural control.

Protein expression of GRIM-19-overexpression was evaluated using western blotting analysis (Fig. 3A), compared to the control group, protein expressions of GRIM-19 in GRIM-19-overexpression GC-2 spd cells were significantly increased (Fig. 3B), moreover, the expression of mRNA of GRIM-19 in GRIM-19-overexpression GC-2 spd cells were significantly increased (Fig. 3C). Protein expression of GRIM-19-silenced was evaluated using western blotting analysis (Fig. 3D), relative to the control group, protein expressions of GRIM-19 in GRIM-19-silenced GC-2 spd cell were significantly decreased (Fig. 3E), moreover, the expression of mRNA of GRIM-19 in GRIM-19-silenced GC-2 spd cell were significantly decreased (Fig. 3F). These results suggest that GRIM-19 overexpression and silencing cell lines were successfully constructed.

### GRIM-19 regulated GC-2 spd cell proliferations

**Figure 4:**
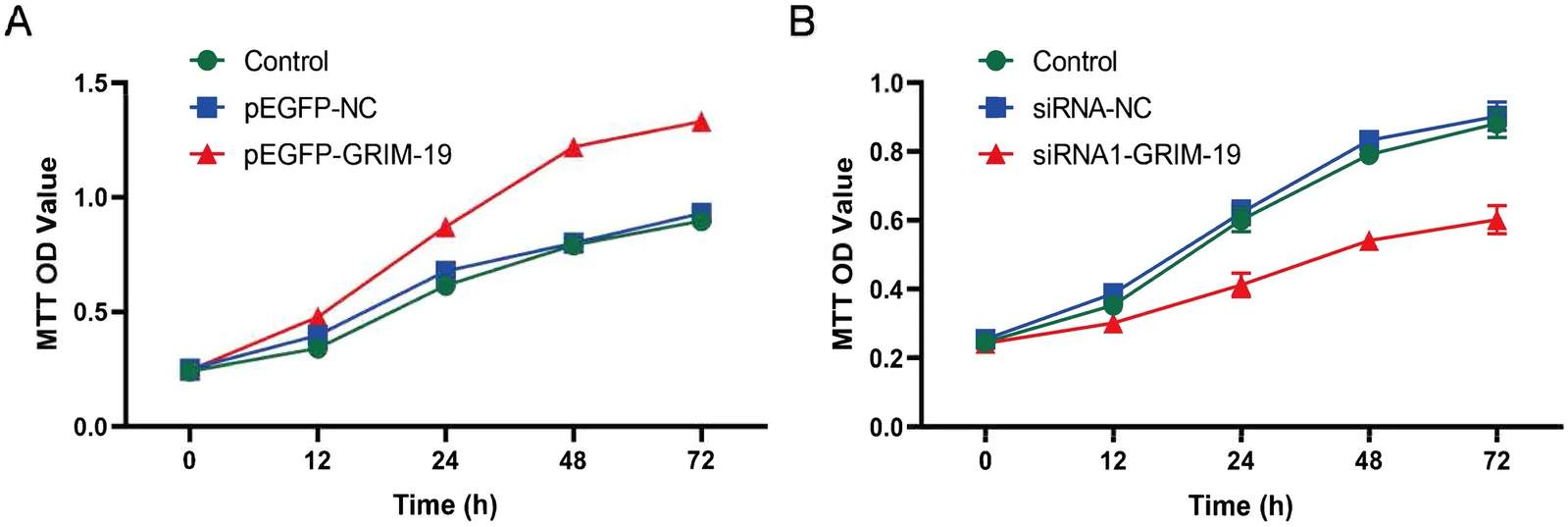
MTT measurement of cell proliferation. (**A**) Cell proliferations of pEGFP-NC and pEGFP-GRIM-19 cells at 0 h, 12 h, 24 h, 48 h, and 72 h after inoculation (0.478 ± 0.032vs.0.342 ± 0.023, 0.872 ± 0.034 vs 0.615 ± 0.032, 1.221 ± 0.021 vs 0.792 ± 0.022, 1.332 ± 0.041 vs 0.898 ± 0.035, P < 0.05). (**B**) Cell proliferations of siRNA-NC and siRNA-GRIM-19 cells at 0 h, 12 h, 24 h, 48 h, and 72 h after inoculation (0.301 ± 0.023 vs 0.353 ± 0.021, 0.372 ± 0.023 vs 0.601 ± 0.035, 0.443 ± 0.020vs 0.691 ± 0.021, 0.552 ± 0.041 vs 0.822 ± 0.039, P < 0.05).

The proliferations of pEGFP-GRIM-19, pEGFP-NC, siRNA-GRIM-19 and siRNA-NC cells lines were evaluated by the MTT assay. Compared to the pEGFP-NC group, the proliferative capacity of GC-2 spd cells in the pEGFP-GRIM-19 group were significantly increased at 12 h, 24 h, 48 h and 72 h after inoculation (0.478 ± 0.032vs.0.342 ± 0.023, 0.872 ± 0.034 vs 0.615 ± 0.032, 1.221 ± 0.021 vs 0.792 ± 0.022, 1.332 ± 0.041 vs 0.898 ± 0.035, *P* < 0.05) (Fig. 4A). Compared to the siRNA-NC group, the proliferation capacity of GC-2 spd cells in the siRNA-GRIM-19 group were markedly decreased at 12 h, 24 h, 48 h and 72 h after inoculation (0.301 ± 0.023 vs 0.353 ± 0.021, 0.372 ± 0.023 vs 0.601 ± 0.035, 0.443 ± 0.020vs 0.691 ± 0.021, 0.552 ± 0.041 vs 0.822 ± 0.039, *P* < 0.05) (Fig. 4B). The results suggested that GRIM-19 could regulate the proliferation of GC-2 spd cells.

### GRIM-19 regulated GC-2 spd cell apoptosis

**Figure 5:**
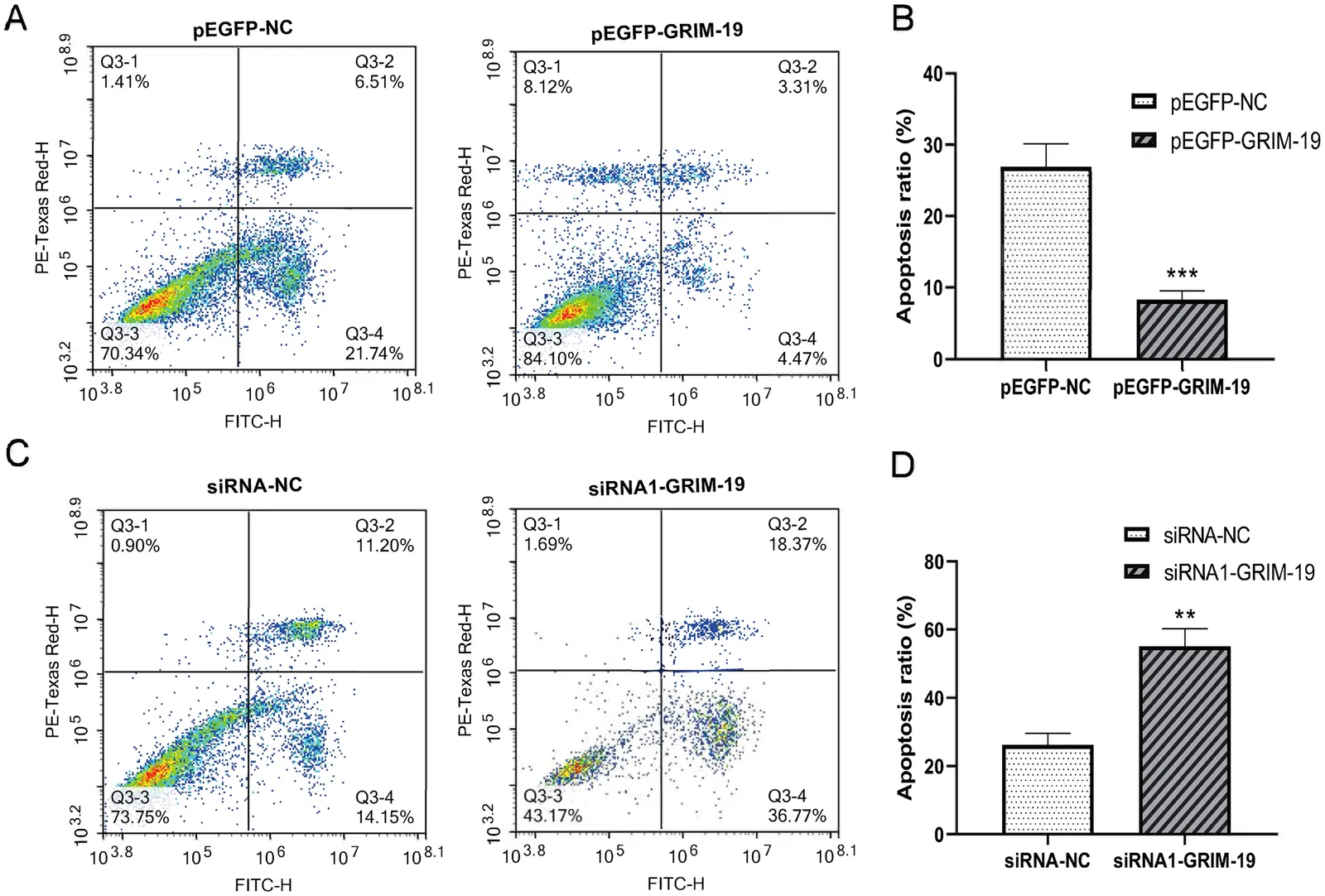
Apoptosis analyzed by flow cytometry. (**A**) The apoptosis of GC-2 spd cell induced by pEGFP-NC and pEGFP-GRIM-19 cells (the figure is divided into four quadrants, the upper left /Q1 represents the distribution of fragmented cells. The upper right/Q2 represents the distribution of late apoptotic cells, the lower left/Q3 represents the distribution of living cells, and the lower right/Q4 represents the distribution of early apoptotic cells). (**B**) Percentage of apoptosis in Q2 + Q4 quadrant of pEGFP-NC and pEGFP-GRIM-19 cells (26.8 ± 3.5% vs 9.7 ± 1.4%, P < 0.001). (**C**) The apoptosis of GC-2 spd cell induced by siRNA-NC and siRNA-GRIM-19 cells. (**D**) Percentage of apoptosis in Q2 + Q4 quadrant of siRNA-NC and siRNA-GRIM-19 cells (24.5 ± 3.2% vs 52.2 ± 7.3%, P < 0.01). ** p < 0.01, *** p < 0.001.

The apoptosis of pEGFP-GRIM-19 and pEGFP-NC cells were evaluated by flow cytometry (Fig. 5A). Compared to the pEGFP-NC group, the apoptosis rate of GC-2 spd cells in the pEGFP-GRIM-19 group was significantly reduced (26.8 ± 3.5% vs 9.7 ± 1.4%, *P* < 0.001) (Fig. 5B). The apoptosis of siRNA-GRIM-19 and siRNA-NC cells were evaluated by flow cytometry (Fig. 5C). Compared to the siRNA-NC group, the apoptosis rate of GC-2 spd cells in the siRNA-GRIM-19 group was significantly increased (24.5 ± 3.2% vs 52.2 ± 7.3%, *P* < 0.01) (Fig. 5D). The results suggested that GRIM-19 could regulate the apoptosis of GC-2 spd cells.

### GRIM-19 regulated GC-2 spd cell migration

**Figure 6:**
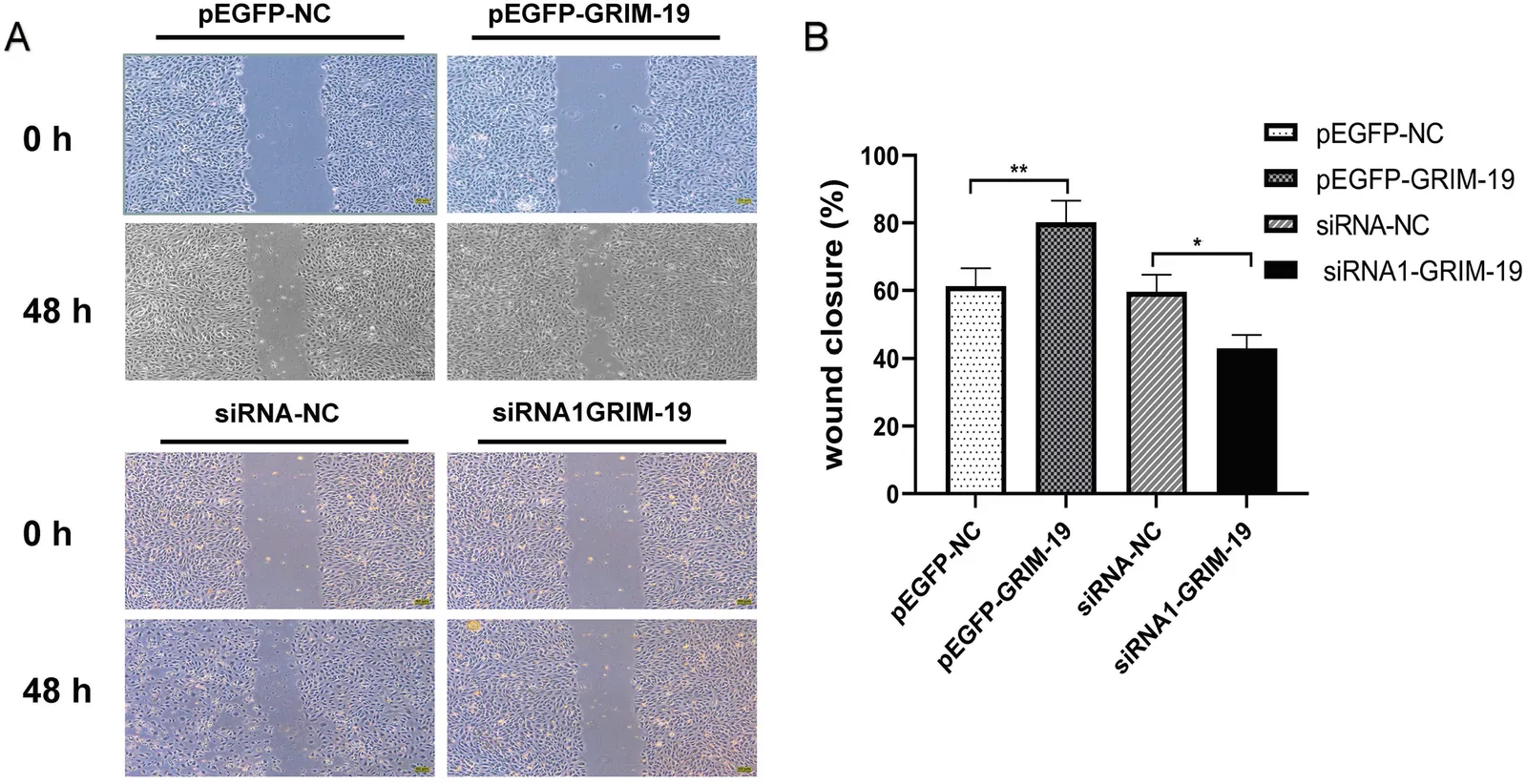
Scratch test to assess the migration of GC-2 spd cell at 0 h and 48 h. The percent of migrated pEGFP-NC and pEGFP-GRIM-19 cells (60.2 ± 5.3% vs 79.8 ± 6.1%, P < 0.01), the percent of migrated siRNA-NC and siRNA-GRIM-19 cells (57.6 ± 5.1% vs 43.2 ± 4.6%, P < 0.05).

Migrating cells in pEGFP-GRIM-19, pEGFP-NC, siRNA-GRIM-19 and siRNA-NC groups were evaluated by the scratch test (Fig. 6A). Compared to the pEGFP-NC group, the migration capacity of GC-2 spd cells in the pEGFP-GRIM-19 group were significantly increased (60.2 ± 5.3% vs 79.8 ± 6.1%, *P* < 0.01), compared to the siRNA-NC group, the migration capacity of GC-2 spd cells in the siRNA-GRIM-19 group were markedly decreased (57.6 ± 5.1% vs 43.2 ± 4.6%, *P* < 0.05) (Fig. 6B). The results suggested that GRIM-19 could regulate the migration of GC-2 spd cells.

## Discussion

The pathogenesis of asthenozoospermia is complicated, the pathogenesis remains unclear, which causes considerable difficulty in developing an effective treatment^17^. As is well known, the human sperm is a special cell that uses flagellum for locomotion and has the ability to fertilize the female egg. Therefore, sperm motility is an important reference index to evaluate male fertility^18^. Anatomical studies of the female reproductive system show that the average distance of the human sperm to complete the fertilization process is about 19cm^19^. Therefore, it is essential to have a certain number of forward motile sperms to be able to conceive. Sperm motility is powered by mitochondria, which are important cellular organelles, it has been shown that mitochondrial respiratory enzyme activity is directly correlated with sperm motility, the mitochondria continuously produce adenosine triphosphate through glycolysis and oxidative phosphorylation to provide energy for sperm activities^20^. As a result, once the mitochondrial structure and function are impaired, the vitality of sperm is reduced.

GRIM-19 is an apoptosis-related gene that widely exists in the cytoplasm and nucleus of cells, it is the basic functional subunit of mitochondrial NADH dehydrogenase complex, and maintains the normal functions of oxidative phosphorylation of respiratory chain in the mitochondrial intima, it is involved in cell proliferation, apoptosis and regulation of various signaling pathways^21,22^. Previous studies showed GRIM-19 is closely associated with oxidative stress injury, collapse of the MMP and production of ROS increased in GC-2 spd cell^21^. Mitochondrial ROS production and aggregation has also been shown to be increased in cells of gastric cancer patients with GRIM-19 deletion, resulting in abnormal mitochondrial functions and activation of the intracellular antioxidant system. We found that GRIM-19 is closely related to the occurrence of asthenozoospermia. The immunofluorescence showed that GRIM-19 was mainly distributed in the head and middle of sperm, and the mRNA and protein levels of GRIM-19 in the asthenozoospermia group were significantly lower than the normal control group. These findings prompted us to further investigate whether GRIM-19 involved in the regulation and functions of GC-2 spd cell. In this study, we used an MTT assay, flow cytometry and a scratch assay confirmed that GRIM-19 can promotes GC-2 spd cell proliferation, migration and reduces apoptosis. On the whole, the mitochondria play an important role in human sperm motility and GRIM-19 is involved in human sperm motility by affecting mitochondrial functions.

Studies have shown that down-regulated GRIM-19 leads to the collapse of mitochondrial membrane potential and an increase in reactive oxygen species levels, these high ROS levels can eventually cause damage to nucleic acids and proteins in cells^15^. Zhang et al.^23^ found that overexpressed GRIM-19 significantly suppressed hypoxia-induced accumulation of ROS and HIF1α, and inhibited vascular endothelial growth factor (VEGF) production as well as STAT3 activation. Bonanno et al.^3^ found that ROS is associated with sperm abnormalities, and its levels were increased in the semen of asthenospermia patients. GRIM-19 plays an important role in cell electron transfer, down-regulation of GRIM-19 leads to the increased of ROS levels, the change in ROS concentration is closely related to sperm motility, capacitation and acrosomal reaction^24^. Also, in addition, the association between GRIM-19 and asthenospermia is also reflected in STAT3, GRIM-19 is a STAT3-specific inhibitory protein, it can directly bind to the STAT3 transcription factor in the cytoplasm and block it from entering the nucleus, thereby down-regulating its transcriptional activity in the nucleus^25^. The STAT3 protein is abundant in the membrane and flagella of the sperm head. It is widely expressed in the dense fibrous structure outside the sperm and is closely related to sperm functions^26^. In the mitochondria, STAT3 mediates oxidative damage responses, regulates apoptosis and plays an important role in non-nuclear transcription. In vitro, STAT3 inhibitors were exposed to active sperms, and it was found that mitochondrial membrane depolarization increased, ROS increased, adenosine triphosphate (ATP) production decreased and sperm motility decreased^27,28^. GRIM-19 might be involved in regulation of sperm motility. It can reduce sperm cell damage and improves sperm motility by regulating the expressions of relevant signaling pathways and protein genes.

In recent years, with rapid advances in genetics and molecular biology, it has been realized that the occurrence of asthenospermia is also associated with oxidative stress damage^29^. The study revealed that there is a close relationship between Grim-19 and oxidative stress damage, collapsed MMP and increased ROS levels were found in GC-2 spd cell after GRIM-19 silencing. Mitochondrial ROS production and aggregation were found to be increased in cells of gastric cancer patients with grim-19 deficiency, leading to abnormal mitochondrial functions and activation of intracellular antioxidant systems^30^. These findings suggest that GRIM-19 plays an important role in regulation of sperm motility in asthenospermia patients through oxidative stress. In 1946, Tosic J and Walton A first revealed that oxidative stress affects the metabolism, motor ability and causes structural and functional defects of the bovine sperm^31^. Similarly, excessive ROS caused by oxidative stress can cause mitochondrial dysfunction. One study showed increased ROS levels and activation of related pathways inhibits ATP production and decreases sperm motility, however, the specific mechanisms are unclear^10^. The mitochondria, which are the main sources of ATP required for sperm motility, are extremely sensitive to ROS, ROS is a product of aerobic metabolism, an appropriate amount of ROS can regulate sperm motility under physiological conditions, however, high ROS levels can lead to oxidative damage to sperm DNA and mitochondrial plasma membrane peroxidation, as a result, serious interference occurs in sperm movement, acrosome reactions and sperm-egg combination^32,33^.

In additional, Studies have shown that there is a positive correlation between sperm motility and mitochondrial membrane potential (MMP), and sperm capacitation is closely related to MMP, measurement of MMP is also used as an important sensitive indicator for sperm mitochondrial function^34^, it is essential for maintaining oxidative phosphorylation and ATP synthesis in mitochondria to maintain normal mitochondrial function. At normal levels, MMP reflects good sperm energy metabolism and ATP synthesis, however, a decrease or disappearance of MMP indicates impaired sperm energy metabolism and ATP synthesis^35^. A study showed that MMP is crucial for ATP synthesis, and mitochondrial dysfunction leads to anoxic cell death and mitochondrial dysfunction^32^. In addition, the higher MMP and ROS production result in oxidative stress, which negatively impacts mitochondrial homeostasis^36^, this aspect may be clarified in the near future through further functional studies (Supplementary file).

In conclusion, GRIM-19 is closely related to the occurrence of asthenozoospermia, GRIM-19 overexpression promotes GC-2 spd cell proliferation and migration and reduces apoptosis, while GRIM-19-silenced reduces GC-2 spd cell proliferation and migration and increased apoptosis. The findings of this study are important for a comprehensive understanding of asthenozoospermia and its clinical treatment.

## Supplementary Information

Supplementary Information.

## Data Availability

The datasets presented in this study are available from the corresponding author on reasonable request.
