# Supplementary figures and images for: GRIM-19 in asthenozoospermia regulates GC-2 spd cell proliferation, apoptosis and migration
Source: Sci Rep. 2023 Feb 22;13:3106. doi: 10.1038/s41598-023-29775-7 (PMC9947114; doi:10.1038/s41598-023-29775-7)

Figure 2B

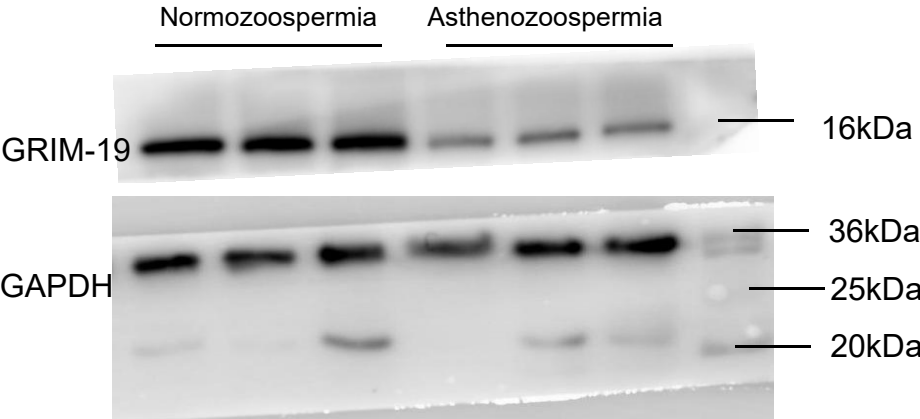

Figure 3A

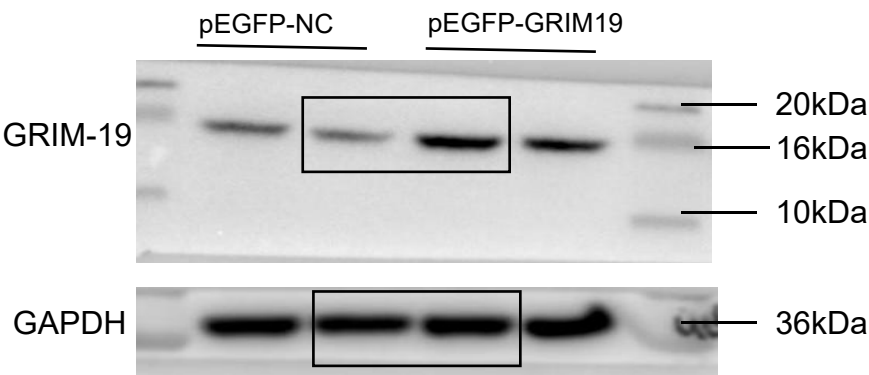

Figure 3D

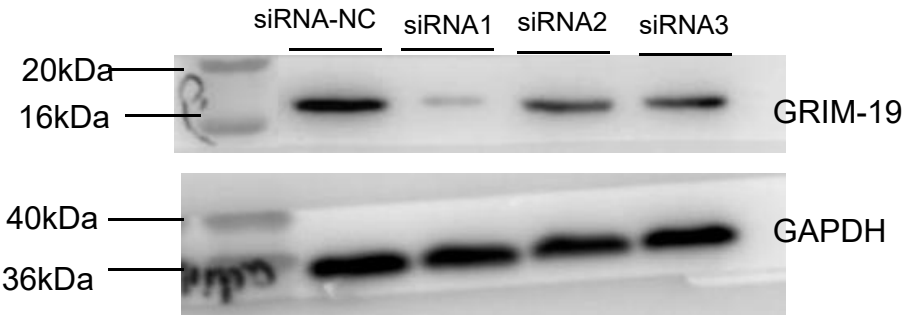

Fuller-length

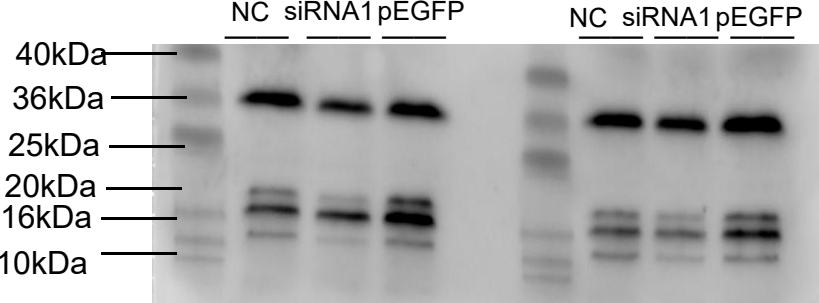

Supplement: Supplementary file 1 — Supplementary Information. [file 41598_2023_29775_MOESM1_ESM.pdf]
